# The clinical value of analyzing predictive models for gastric function and gastric organic lesions

**DOI:** 10.3389/fendo.2026.1852014

**Published:** 2026-06-15

**Authors:** Youlin Song, Yi Hu, Kang Wang, Yanhua Zhu, Yang Wang, Tao Liu, Lihao Wu, Xingxiang He, Lei Wu

**Affiliations:** 1 Department of Gastroenterology/Microbiota Medicine, The First Affiliated Hospital of Guangdong Pharmaceutical University, Guangzhou, China; 2 Department of Respiratory and Critical Care Medicine, The First Affiliated Hospital of Army Medical University, Chongqing, China; 3 Department of Information; Information Section; Information Center, The First Affiliated Hospital of Guangdong Pharmaceutical University, Guangzhou, China; 4 School of Life and Health Technology, Dongguan University of Technology, Dongguan, China; 5 Department of Medical Record Statistics, The First Affiliated Hospital of Guangdong Pharmaceutical University, Guangzhou, China

**Keywords:** clinical value, gastric function, gastric organic lesions, prediction model, serum markers

## Abstract

**Objective:**

To explore the clinical value of the predictive models for gastric function and gastric organic lesions.

**Methods:**

Clinical data from 1,677 patients with digestive tract-related symptoms at the First Affiliated Hospital of Guangdong Pharmaceutical University between June 2015 and March 2023 were selected, including questionnaire responses, serum gastric function tests, gastroscopy, and gastroscopic pathology data. The above data were input into a gastric function prediction model based on question-and-answer [GFPSBQA] V1.0 and a gastric organic lesion prediction model [GDPSIQSM] V1.0, both of which integrated questionnaires and serum markers. The model’s output results were compared with the results of laboratory tests and gastroscopy (including pathology), and the model’s accuracy was analyzed.

**Results:**

The sensitivity of the gastric function prediction model was 91.16%, the specificity was 39.18%, the positive predictive value was 83.27%, the negative predictive value was 57.14%, the area under the receiver operating characteristic curve (AUC) was 0.702, and the Youden index was 0.303. The sensitivity of the gastric organic lesion prediction model was 89.55%, the specificity was 33.22%, the positive predictive value was 70.86%, the negative predictive value was 63.67%, the AUC was 0.672, and the Youden index was 0.228.

**Conclusion:**

Both the gastric function prediction model and the gastric organic lesion prediction model demonstrate high sensitivity and good predictive performance, and may have potential clinical applicability.

## 1 Introduction

China is one of the regions with a high incidence of gastric cancer. Although its incidence and mortality rates have shown a downward trend in recent years, the overall disease burden remains significantly higher than the global average (1). Gastric cancer typically undergoes a pathological progression from chronic inflammation to atrophy, intestinal metaplasia, and intraepithelial neoplasia; this “inflammation-to-cancer” continuum provides a critical window for early screening and risk intervention. Therefore, establishing efficient and scalable screening strategies is crucial for achieving early diagnosis and treatment of gastric cancer.

Currently, the four serum gastric function markers—pepsinogen I (PGI), pepsinogen II (PGII), gastrin 17 (G17), and Helicobacter pylori (H. pylori) antibodies—serve as important indicators for detecting gastric diseases (2–5); The H. pylori positivity rate in patients with gastric mucosal lesions increases with the severity of the lesions, while PGⅠ and PGⅠ/PGⅡ levels change in accordance with the severity of the lesions (6, 7). Pathological examination of endoscopic biopsy specimens is the “gold standard” for diagnosing gastric organic lesions (8), but it is difficult to apply universally for screening for gastric organic lesions. Screening and early diagnosis and treatment are universally recognized worldwide as effective means of reducing cancer incidence and mortality (9).

In recent years, although various gastric cancer risk prediction models have been developed both domestically and internationally, most have focused on cancer-related endpoints and relied on single data sources (such as clinical indicators or epidemiological factors). There remain shortcomings in integrating multidimensional information and guiding stratified screening pathways (10–12). Based on these considerations, the present study clinically validated the previously developed [Gastric Function Prediction System Based on Question and Answer (GFPSBQA)] V1.0 (13) and [Gastric Disease Prediction System Integrating Question and Serological Markers (GDPSIQSM)] V1.0 (14) within a tiered and progressive screening framework. Specifically, GFPSBQA was initially applied for questionnaire-based population screening, followed by GDPSIQSM integrating serological indicators for secondary risk reassessment, thereby establishing a stepwise “questionnaire–serology–endoscopy” screening pathway (15). Unlike existing gastric cancer prediction tools that mainly rely on single-dimensional indicators or focus primarily on cancer-related outcomes, this framework integrates multidimensional risk information and extends risk assessment to broader gastric functional abnormalities and organic lesions. This strategy aims to facilitate risk stratification prior to endoscopy, improve screening efficiency, and optimize healthcare resource allocation. Therefore, this study systematically evaluated the clinical performance and practical applicability of the aforementioned models in a real-world patient population, with the aim of providing a feasible approach for the early screening of gastric diseases.

## 2 Materials and methods

### 2.1 Development of the GFPSBQA and GDPSIQSM models

Prior to this study, the GFPSBQA and GDPSIQSM models were jointly developed by the First Affiliated Hospital of Guangdong Pharmaceutical University and the School of Computer Science at Guangdong University of Technology (15). Based on individual items from the gastric function questionnaire, weight analysis was performed using principal component analysis (PCA), the entropy weighting method, and the analytic hierarchy process (AHP) to identify key variables, low-correlation variables, and negatively contributing variables. According to the weighting results, different feature sets were constructed, including high-quality questionnaire-derived features (consisting of key variables only) and low-quality questionnaire-derived features (comprising key variables combined with low-correlation variables), which were subsequently transformed into feature vectors for model development. Based on these feature engineering procedures, the GFPSBQA model was established. Subsequently, questionnaire-derived gastric function data were integrated with serological gastric function biomarkers (four-item blood test indicators). The dataset was randomly expanded and partitioned into training, testing, and validation cohorts, followed by a 10-fold cross-validation procedure. Multiple machine-learning algorithms, including support vector machine (SVM), random forest (RF), and decision tree (DT), were applied to classify and optimize features derived from the large-scale gastric function questionnaire dataset. The testing and validation cohorts were separately used to evaluate the learning performance of the developed model on the training dataset and its generalizability to independent unseen data. Furthermore, a feature-combination analytical framework was established to statistically assess the distributional trends of different gastric function abnormality feature combinations, thereby leading to the development of the GDPSIQSM model. The GFPSBQA and GDPSIQSM systems were developed prior to the present study using historical clinical datasets through training, testing, and internal validation procedures. Although a limited proportion of early cases may overlap with the present cohort due to the continuous accumulation of clinical data, a substantial number of subsequently enrolled patients were newly entered after model establishment and served as a real-world clinical validation population. The current study therefore primarily reflects the practical performance of the established systems in an expanded clinical cohort. In the application GFPSBQA For the prediction of gastric function, definitions of normal and abnormal gastric function are provided in Appendix 2; In the application GDPSIQSMF or the prediction of gastric organic lesions, definitions of normal and abnormal gastroscopy (including pathology) results are provided in Appendix 3.”Abnormal” gastroscopy (including pathology) was defined based on the clinical significance of lesions and the need for further evaluation or management. Mild-to-moderate chronic non-atrophic gastritis without additional pathological findings was classified as normal, whereas severe chronic non-atrophic gastritis and clinically relevant upper gastrointestinal lesions (e.g., reflux esophagitis or duodenal ulcer) were classified as abnormal due to their greater clinical relevance and potential need for intervention.

### 2.2 Clinical application process for the GFPSBQA and GDPSIQSM models

**Figure 1:**
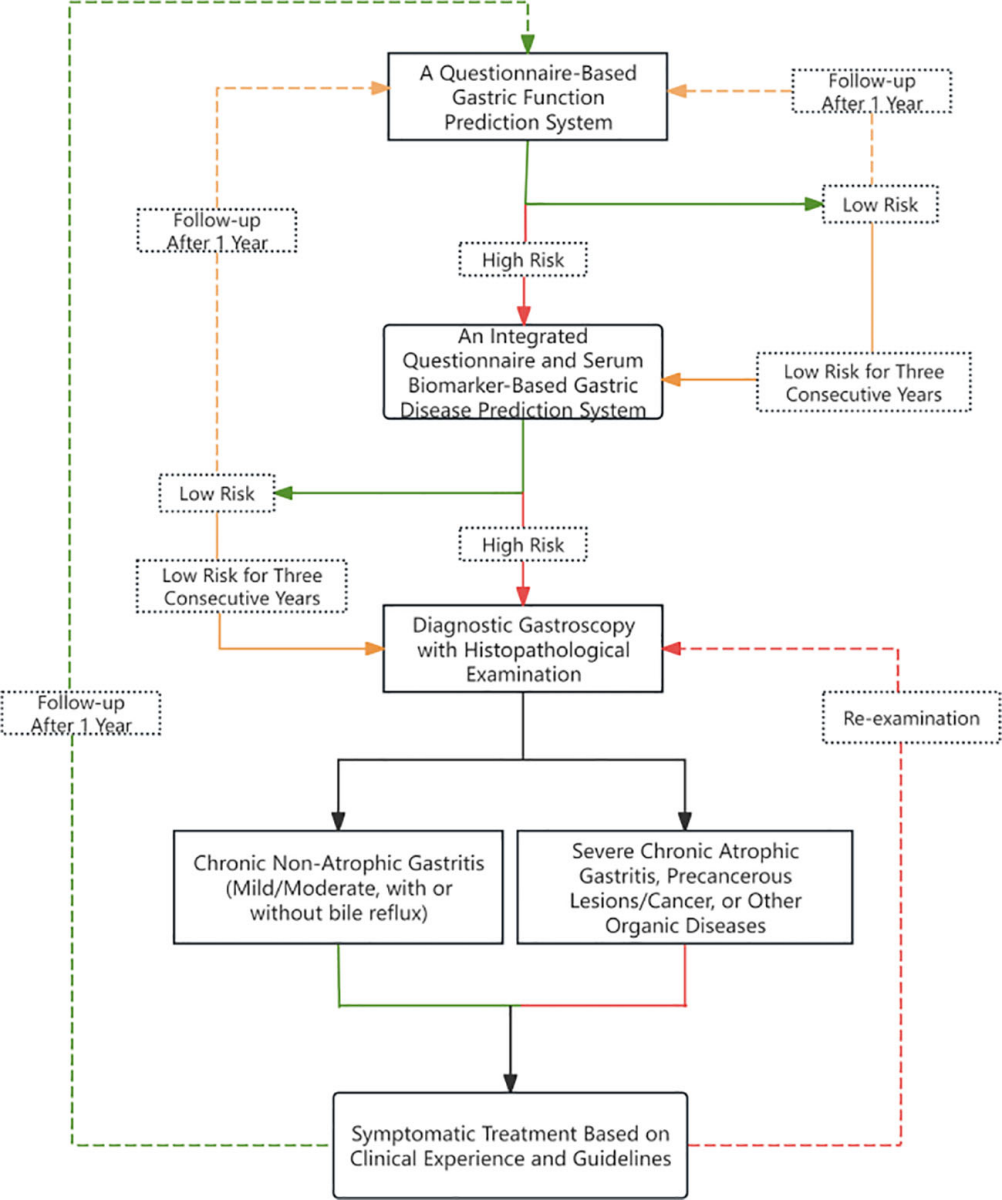
Flowchart of the stepwise screening model for gastric function and organic lesions.

The GFPSBQA and GDPSIQSM models together form a stepwise screening model divided into two stages. First, questionnaire screening using the GFPSBQA model is conducted among the patient population to predict gastric function risk. For those classified as low-risk, a questionnaire screening is repeated after one year. If the GFPSBQA model indicates low risk for three consecutive years, blood testing for the four gastric function markers is still recommended, followed by screening using the GDPSIQSM model. If the classification is high-risk, blood testing for the four gastric function markers is performed, followed by the next step of screening using the GDPSIQSM model. For individuals classified as low-risk by the GDPSIQSM model, the GFPSBQA model screening should be repeated after one year; if the GDPSIQSM model indicates low risk for three consecutive years, a detailed gastroscopy including histopathological examination is still recommended. If the GDPSIQSM model indicates high risk, a detailed gastroscopy including a biopsy should be performed. Following the detailed gastroscopy and biopsy, appropriate management should be determined based on the guidelines and the flowchart for the stepwise screening model of gastric function and gastric organic lesions (**Figure 1**).

### 2.3 Study population

Patients admitted to Guangdong Pharmaceutical University First Affiliated Hospital, Foshan Traditional Chinese Medicine Hospital, Nanxiong Traditional Chinese Medicine Hospital, and other hospitals between June 2015 and March 2023 for digestive symptoms were enrolled in this study, with a total of 1,677 cases. Inclusion Criteria: (1) Age ≥ 18 years; (2) All patients underwent testing for serum markers including pepsin, gastrin-17, and HP antibodies; (3) All patients underwent gastroscopy and histological examination; (4) All patients signed informed consent forms. Exclusion Criteria: (1) Patients with mental disorders or other conditions preventing cooperation with the study; (2) Patients with a history of other malignant tumors.

### 2.4 Research methods

(1) Application of the GFPSBQA Model: Data collection was conducted via face-to-face interviews by trained personnel. Participants first completed a paper-based questionnaire (see Appendix 1), and the information was subsequently entered into the GFPSBQA model. (2) Serum Gastric Function Panel Testing: Blood samples were collected for PGI, PGII, G-17, and HP antibody testing (using ELISA kits and instruments from Biohan). (3) Application of the GDPSIQSM Model: A designated staff member entered the results of the four gastric function parameters into the GDPSIQSM model. (4) Gastroscopy and Pathological Examination: A specialist physician performs gastroscopy and pathological examination on the patient according to standardized protocols. The gastroscopist is highly skilled in performing gastroscopy and is unaware of the patient’s GFPSBQA and GDPSIQSM model assessment results. Prior to the gastroscopy, if the patient consents to mucosal biopsy and provides a signed consent form, a routine mucosal biopsy is performed. Otherwise, no mucosal biopsy is performed. For patients who consent to pathological examination, the biopsy criteria for gastroscopy are as follows: If suspicious mucosal lesions are identified endoscopically, a biopsy specimen of the suspicious mucosa is obtained using disposable biopsy forceps after observation under magnified and stained endoscopy. If no potential mucosal lesions are identified endoscopically, a biopsy is routinely performed 2 cm from the pylorus on the greater curvature side of the gastric antrum.

### 2.5 Statistical methods

Statistical analysis was performed using Excel 2010 and SPSS 23.0 software. Quantitative data with a normal distribution were described as mean ± standard deviation, and intergroup comparisons were performed using the t-test; quantitative data with a non-normal distribution were described as median and interquartile range, and intergroup comparisons were performed using the Mann-Whitney U test. Qualitative data were described as frequency and percentage, and intergroup comparisons were performed using the chi-square test. The practical performance of the model was evaluated using indicators such as sensitivity, specificity, the Youden index, and agreement rate. A p-value < 0.05 was considered statistically significant.

## 3 Results

### 3.1 General characteristics of all patients

#### 3.1.1 Patient demographics

From June 2015 to March 2023, a total of 1,677 patients with complete questionnaire data, serum gastric function test results, and endoscopic findings (including pathology) were included in the study. Among them, 801 were male (47.8%) and 876 were female (52.2%), with a male-to-female ratio of 0.9:1.0.There were 274 patients (16.3%) aged 40 years or younger and 1,403 patients (82.7%) aged over 40 years (**Table 1**):

**Table 1:** Demographic data (n=1677).

Variable	Category	Number of cases	Percentage (%)
Gender	Male	801	47.8
	Female	876	52.2
Age	≤40 years old	274	16.3
	>40 years	1,403	82.7
Place of residence	Towns and rural areas	517	30.8
	Urban	1,160	69.2
Educational attainment	Elementary school and below	300	17.9
	Junior high and high school	685	40.8
	College and above	692	41.3
Occupation	White-collar workers	695	41.4
	Laborers	327	19.5
	Farmers	232	13.8
	Students	2	0.1
	Freelancer	54	3.2
	Unemployed and underemployed	367	21.9
Everyday language	Mandarin	781	46.6
	Cantonese	473	28.2
	Hakka	273	16.3
	Chaozhou dialect	135	8.1
	Other	15	0.9
Family history of cancer (esophageal, gastric, or colorectal)	None	1,547	92.2
	Yes	130	7.8
High-salt diet (daily salt intake greater than 10 grams, equivalent to one capful of a soda bottle)	No	1,383	82.5
	Yes	294	17.5
Intake of pickled foods	Occasionally (less than three times a week)	1,525	90.9
	Frequently (at least three times a week)	142	8.5
Intake of fried and smoked foods	Occasionally (less than three times a week)	1566	93.4
	Frequently (at least three times a week)	111	6.6
Fresh fruit intake	Occasionally (less than three times a week)	942	56.2
	Frequently (at least three times a week)	735	43.8
Fresh vegetable intake	Occasionally (less than three times a week)	523	31.2
	Frequently (at least three times a week)	1,154	68.8
Tea consumption	Occasionally (less than three times a week)	510	30.4
	Frequently (at least three times a week)	1,167	69.6
Smoking history	None	1286	76.7
	Yes	391	23.3
History of alcohol consumption	None	1,429	85.2
	Yes	248	14.8
Eating speed	Fast (meal duration less than 10 minutes)	482	28.7
	Slow (meal duration of 10 minutes or more)	1,195	71.3
Diabetes (FBS ≥ 7.0 mmol/L or 126 mg/dL)	Unknown	232	13.8
	No	1,338	79.8
	Yes	107	6.4
Hypertension (BP ≥ 140/90 mmHg or taking antihypertensive medication)	Unknown	254	15.1
	No	1,193	71.1
	Yes	230	13.7
Hypertriglyceridemia (TG ≥ 1.7 mmol/L or taking lipid-lowering medication)	Unknown	314	18.7
	No	1,104	65.8
	Yes	259	15.4

#### 3.1.2 Distribution of gastroscopic diagnoses

Among the 1,677 study subjects included, 1,081 had positive endoscopic findings (including pathology), yielding a positive detection rate of 64.46%. The most common diagnoses were gastric mucosal erosion, gastric polyps, and gastric mucosal atrophy, with 297, 286, and 144 cases, respectively; accounting for 17.71%, 17.05%, and 8.59% of the total cases, respectively. Other gastric diagnoses included intestinal metaplasia (69 cases), gastric ulcer (65 cases), gastric intraepithelial neoplasia (52 cases), gastric cancer (21 cases), and gastrointestinal stromal tumor (4 cases). Duodenal diagnoses included 28 cases of duodenal polyps, 136 cases of duodenal bulb ulcers, 51 cases of reflux esophagitis, 13 cases of Barrett’s esophagus, 27 cases of esophageal varices, and 23 cases of hiatal hernia (**Table 2**).

**Table 2:** Specific endoscopic diagnoses in patients with positive endoscopic findings (including pathology).

Site of gastroscopy	Gastroscopy vs. pathological diagnosis	Number	Proportion (%)
Stomach			
	Erosion	297	17.71
	Ulcer	65	3.88
	Atrophy	144	8.59
	Intestinal metaplasia	69	4.11
	Intraepithelial neoplasia	52	3.10
	Cancer	21	1.25
	Polyp	286	17.05
	Stromal tumor	4	0.24
Duodenum			
	Polyp	28	1.67
	Ulcer	136	8.11
Esophagus			
	Reflux esophagitis	51	3.04
	Barrett’s esophagus	13	0.78
	Hiatal hernia	23	1.37
	Esophageal varices	27	1.61

### 3.2 Group patients based on the conclusions from the GFPSBQA and GDPSIQSM models, and compare differences in baseline characteristics between the two groups

#### 3.2.1 Based on the results of the GFPSBQA model, patients were classified into a low-risk group and a high-risk group for gastric function. Differences in the baseline characteristics between the two groups

Among the 1,677 study subjects, 266 were in the low-risk gastric function group and 1,411 were in the high-risk gastric function group. A comparative analysis of the baseline characteristics between the two groups revealed significant differences (P < 0.05) in place of residence, occupation, intake of pickled foods, intake of fried and smoked foods, intake of fresh vegetables, tea consumption, smoking history, diabetes, hypertension, and hypertriglyceridemia (**Table 3**).

**Table 3:** Demographic characteristics of patients in the low-risk and high-risk groups for gastric function.

Item	Category	Low-risk group for gastric function (n= 266)	(n= 1411)	P
Gender	Male	123 (46.24%)	678 (48.05%)	0.588
	Female	143 (53.76%)	733 (51.95%)	
Age		55.00 (45.75, 63.00)	54.00 (44.00, 63.00)	0.286
Place of residence	City	165 (62.03%)	995 (70.52%)	0.006
	Towns and Rural Areas	101 (37.97%)	416 (29.48%)	
Educational Attainment	College degree or higher	115 (43.23%)	577 (40.89%)	0.477
	High school or below	151 (56.77%)	834 (59.11%)	
Occupational	Farmers, workers	106 (39.85%)	453 (32.10%)	<0.001
	Cadres, clerical staff	122 (45.86%)	573 (40.61%)	
(%)	Students, unemployed, and self-employed	38 (14.29%)	385 (27.29%)	
Family history of cancer (esophageal, gastric, or colorectal)	Yes	23 (8.65%)	107 (7.58%)	0.552
	None	243 (91.35%)	1,304 (92.42%)	
High-salt diet (daily salt intake greater than 10 grams, equivalent to one soda bottle cap)	Yes	53 (19.92%)	241 (17.08%)	0.263
	No	213 (80.08%)	1,170 (82.92%)	
Intake of pickled foods	Occasionally	238 (89.47%)	114 (8.08%)	<0.001
	Frequently	28 (10.53%)	1,297 (91.92%)	
Intake of fried and smoked foods	Occasionally	247 (92.86%)	92 (6.52%)	<0.001
	Frequent	19 (7.14%)	1,319 (93.48%)	
Fresh fruit intake	Occasionally	146 (54.89%)	615 (43.59%)	0.001
	Frequently	120 (45.11%)	796 (56.41%)	
Fresh vegetable intake	Occasionally	80 (30.08%)	443 (31.40%)	0.670
	Frequently	186 (69.92%)	968 (68.60%)	
Tea intake	Occasionally	171 (64.29%)	996 (70.59%)	0.040
	Frequently	95 (35.71%)	415 (29.41%)	
Smoking history	Yes	75 (28.20%)	316 (22.40%)	0.040
	None	191 (71.80%)	1,095 (77.60%)	
History of alcohol consumption	Yes	36 (13.53%)	212 (15.02%)	0.530
	None	230 (86.47%)	1,199 (84.98%)	
Feeding rate	Fast	80 (30.08%)	402 (28.49%)	0.600
	Slow	186 (69.92%)	1,009 (71.51%)	
Diabetes (FBS ≥ 7.0 mmol/L or 126 mg/dL)	Unknown	20 (7.52%)	212 (15.03%)	0.001
	Yes	24 (9.02%)	83 (5.88%)	
	No	222 (83.46%)	1,116 (79.09%)	
Hypertension (BP ≥ 140/90 mmHg or taking antihypertensive medication)	Unknown	17 (6.39%)	237 (16.80%)	<0.001
	Yes	47 (17.67%)	183 (12.97%)	
	No	202 (75.94%)	991 (70.23%)	
Hypertriglyceridemia (TG ≥ 1.7 mmol/L or taking lipid-lowering medication)	Unknown	24 (9.02%)	290 (20.55%)	<0.001
	Yes	50 (18.80%)	209 (14.81%)	
	No	192 (72.18%)	912 (64.64%)	

#### 3.2.2 Differences in baseline characteristics between the low-risk and high-risk groups for gastric organic lesions, as classified by the GDPSIQSM model

Among the 1,677 study subjects included, 311 were in the low-risk group for gastric organic lesions, and 1,366 were in the high-risk group. A comparative analysis of the baseline characteristics between the two groups revealed no significant differences between the low-risk group and the high-risk group for gastric organic lesions in terms of gender, age, place of residence, educational level, family history of cancer, high-salt diet, intake of pickled foods, fried foods, smoked foods, smoked food intake, fresh fruit intake, fresh vegetable intake, tea consumption, smoking history, alcohol consumption history, eating speed, diabetes, hypertension, hypertriglyceridemia, HP (antibody), PGI, and PGI (**Table 4**).

**Table 4:** Demographic characteristics of patients in the low-risk and high-risk groups for gastric organic lesions.

Item	Category	Low-risk group for gastric organic lesions (n=311)	High-risk group for gastric organic lesions (n=1366)	P
Gender	Male	123 (39.55%)	678 (49.63%)	0.001
	Female	188 (60.45%)	688 (50.37%)	
Age		48.00 (38.00, 58.00)	55.00 (46.00, 64.00)	<0.001
Place of residence	City	281 (90.35%)	879 (64.35%)	<0.001
	Towns and rural areas	30 (9.65%)	487 (35.65%)	
Educational attainment	College degree or higher	257 (82.64%)	435 (31.84%)	<0.001
	High school or below	54 (17.36%)	931 (68.16%)	
Occupational	Farmers, workers	43 (13.83%)	516 (37.77%)	<0.001
	Cadres, clerical staff	250 (80.39%)	445 (32.58%)	
	Students, unemployed, and self-employed	18 (5.78%)	405 (29.65%)	
Family history of cancer (esophageal, gastric, or colorectal)	Yes	5 (8.04%)	81 (5.93%)	0.002
	None	306 (98.39%)	1,285 (94.07%)	
High-salt diet (daily salt intake greater than 10 grams, equivalent to one soda bottle cap)	Yes	8 (2.57%)	286 (20.94%)	<0.001
	No	303 (97.43%)	1,080 (79.06%)	
Intake of pickled foods	Occasionally	306 (98.39%)	1,229 (89.97%)	<0.001
	Frequently	5 (1.61%)	137 (10.03%)	
Intake of fried and smoked foods	Occasionally	303 (97.43%)	1,263 (92.46%)	0.001
	Frequently	8 (2.57%)	103 (7.54%)	
Fresh fruit intake	Occasionally	293 (94.21%)	649 (47.51%)	<0.001
	Frequently	18 (5.79%)	717 (52.49%)	
Fresh vegetable intake	Occasionally	309 (99.36%)	214 (15.67%)	<0.001
	Frequently	2 (0.64%)	1,152 (84.33%)	
Tea intake	Occasionally	286 (91.96%)	881 (64.49%)	<0.001
	Frequently	25 (8.04%)	485 (35.51%)	
Smoking history	Yes	43 (13.83%)	348 (25.48%)	<0.001
	None	268 (86.17%)	1,018 (74.52%)	
History of alcohol consumption	Yes	15 (4.82%)	233 (17.06%)	<0.001
Xueq	None	296 (95.18%)	1,133 (82.94%)	
Eating speed	Fast	22 (7.07%)	460 (33.67%)	<0.001
	Slow	289 (92.93%)	906 (66.33%)	
Diabetes (FBS ≥ 7.0 mmol/L or 126 mg/dL)	Unknown	30 (9.65%)	202 (14.79%)	<0.001
	Yes	7 (2.25%)	100 (7.32%)	
	No	274 (88.10%)	1,064 (77.89%)	
Hypertension (BP ≥ 140/90 mmHg or taking antihypertensive medication)	Unknown	25 (8.04%)	229 (16.76%)	<0.001
	Yes	15 (4.82%)	215 (15.74%)	
	No	271 (87.14%)	922 (67.50%)	
Hypertriglyceridemia (TG ≥ 1.7 mmol/L or taking lipid-lowering medication)	Unknown	33 (10.61%)	281 (20.57%)	<0.001
	Yes	19 (6.11%)	240 (17.57%)	
	No	259 (83.28%)	845 (61.86%)	
HP (antibody)	Negative	240 (77.17%)	926 (67.79%)	<0.001
	Positive	71 (22.83%)	440 (32.21%)	
Gastrin 17 (G17)		3.29 (1.51, 10.71)	3.50 (1.37, 11.01)	0.858
Pepsinogen I (PG I)		150.48 (115.91, 211.39)	134.46 (91.41, 207.01)	<0.001
Pepsinogen II (PG II)		8.96 (5.72, 13.22)	10.40 (6.53, 18.75)	<0.001

### 3.3 Prediction model output results compared with blood-based gastric function test results and endoscopy findings (including pathology results)

**Figure 2:**
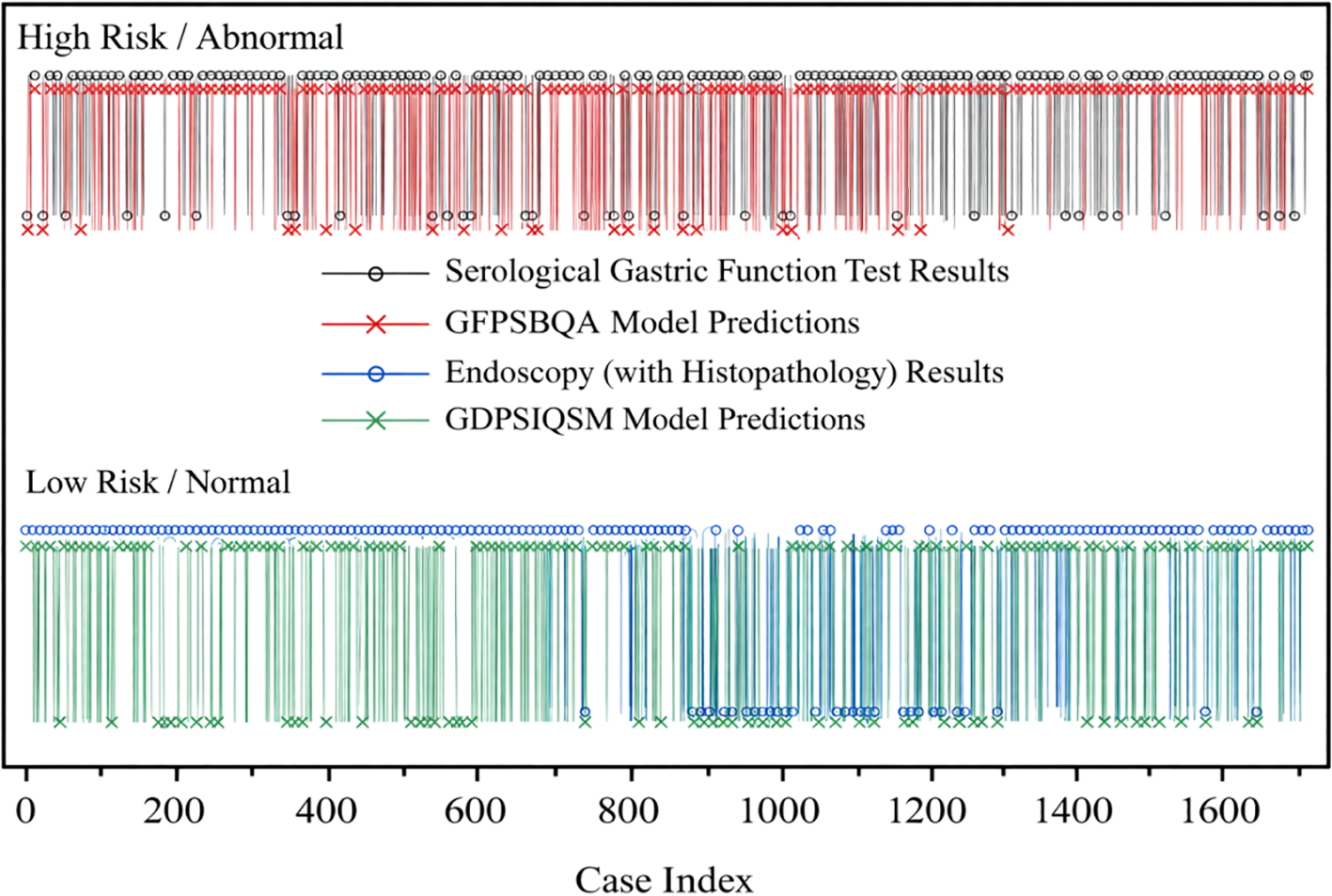
Comparison of model predictions with clinical results.

From June 2015 to March 2023, a total of 1,677 patients were enrolled with complete data for the gastric function prediction questionnaire, blood-based gastric function tests, and gastroscopy (including pathology results). The GFPSBQA model classified 1,411 patients as high-risk and 266 as low-risk;Blood-based gastric function test results were abnormal in 1,289 cases and normal in 388 cases. The GDPSIQSM model classified 1,366 patients as high-risk and 311 as low-risk; endoscopic findings (including pathology) were abnormal in 1,081 cases and normal in 596 cases (**Figure 2**).

#### 3.3.1 Accuracy of GFPSBQA output results

**Figure 3:**
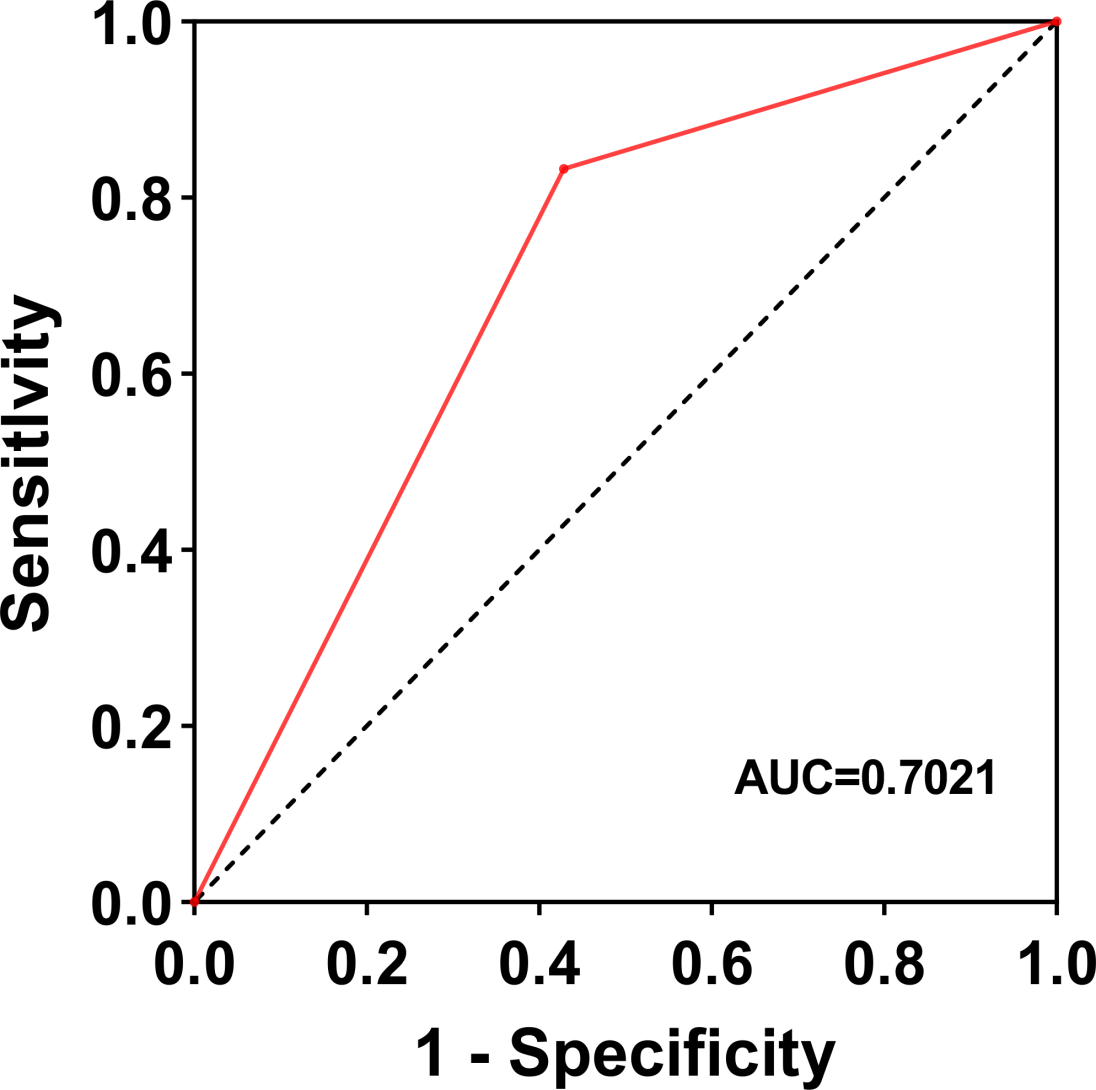
ROC curve for serum gastric function results predicted by the GFPSBQA model.

The GFPSBQA predictive model demonstrated a sensitivity of 91.16%, specificity of 39.18%, positive predictive value of 83.27%, and negative predictive value of 57.14% for predicting blood test results of gastric function; The area under the receiver operating characteristic curve (AUC) was 0.702, with a 95% confidence interval of (0.665, 0.740), and the Youden’s index was 0.303 (**Table 5**; **Figure 3**).

**Table 5:** Predictive performance of the GFPSBQA model for serum gastric function outcomes.

GFPSBQA prediction results	Serological gastric function test results		Sensitivity (%)	Specificity (%)	Youden index
	Abnormal Normal				
High risk	1175	236	91.16	39.18	0.303
Low risk	114	152		
Total	1,289	388		

#### 3.3.2 Accuracy of GDPSIQSM output results

**Figure 4:**
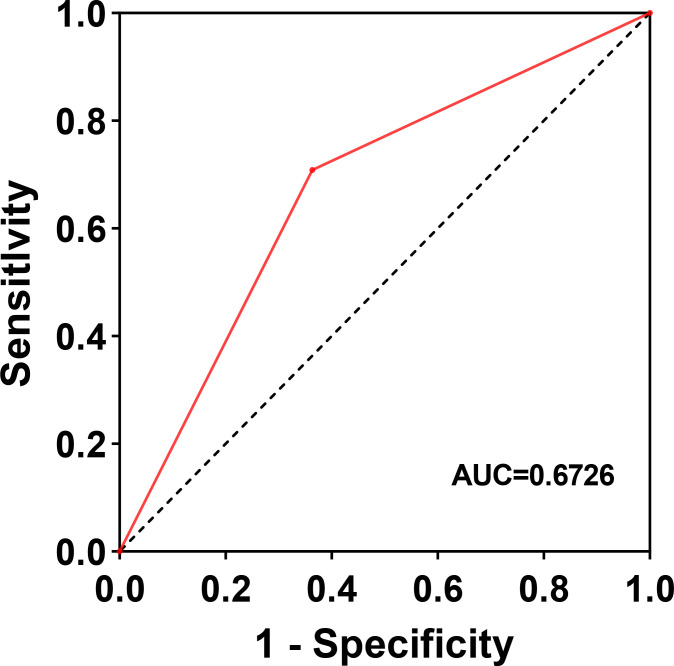
ROC curve for endoscopic findings (including pathology) predicted by the GDPSIQSM model.

The sensitivity of the GDPSIQSM predictive model for predicting gastroscopy (including pathology) results was 89.55%, specificity was 33.22%, positive predictive value was 70.86%, and negative predictive value was 63.67%.the AUC is 0.672, with a 95% confidence interval of (0.639, 0.707), and a Youden index of 0.228 (**Table 6**; **Figure 4**).

**Table 6:** Predictive performance of the GDPSIQSM model for gastroscopy (including pathology) results.

GDPSIQSM prediction results	Gastroscopy (including pathology) results		Sensitivity (%)	Specificity (%)	Youden index
	Abnormal Normal				
High risk	968	398	89.55	33.22	0.228
Low risk	113	198		
Total	1,081	596		

## 4 Discussion

Gastric cancer ranks fifth in incidence and fourth in mortality among malignant tumors worldwide (16). Its occurrence and development are influenced by multiple factors, including dietary patterns, genetic susceptibility, and Helicobacter pylori infection (17–20). Although endoscopy combined with histopathological examination remains the gold standard in the diagnosis of gastric organic lesions (21), its application in large-scale screening is constrained by practical factors such as uneven resource allocation and insufficient patient compliance (22). Therefore, how to improve accessibility while maintaining screening sensitivity has become an urgent issue in the field of gastric disease prevention and control. In the early stages of gastric diseases or functional disorders, serum PG levels and Helicobacter pylori infection status are significantly associated with the development of gastric cancer and precancerous lesions. These markers can identify populations at high risk for gastric cancer and precancerous lesions making them valuable for use in health screening populations, and contributing to the prevention of gastric diseases or functional disorders (23). The long-term survival rates and quality of life for early-stage gastric cancer are significantly better than those intermediate- and advanced-stage gastric cancer for. However, the current diagnosis and treatment rate for early-stage gastric cancer in China is less than 10%, far lower than Japan’s 70% and South Korea’s 50% (24–26).

Current mainstream international screening models, such as the Japanese ABC classification system (27) and the Korean risk prediction models (10), respectively emphasize the application of serological markers and epidemiological factors; China has also established a risk scoring system based on indicators such as PG, G-17, and Hp antibodies (28), indicating a close correlation with the staging of histological atrophy/intestinal metaplasia in the risk assessment of gastric precancerous lesions (29). While these models hold some value in identifying high-risk populations for gastric cancer, they still have the following limitations: they focus on gastric cancer as the endpoint, neglecting broader gastric organic lesions; their variable dimensions are relatively limited, lacking integration of multi-source data; and they lack clear stratified screening pathways, making it difficult to directly guide clinical decision-making. Therefore, developing a model that combines multi-dimensional data integration capabilities with guidance on screening pathways holds significant clinical value.

The GFPSBQA and GDPSIQSM models proposed in this study establish a stepwise screening system comprising “questionnaire-based initial screening—serological re-screening—endoscopic detailed examination.” Their core advantages lie in: multidimensional information integration, which combines epidemiological characteristics, lifestyle factors, and serological indicators to enhance the comprehensiveness of risk assessment; a stratified and progressive design that enables risk stratification through different screening levels, thereby helping to optimize screening pathways; strong applicability at the primary care level, with questionnaire-based screening being simple to administer and suitable for implementation in resource-limited regions. From a mechanistic perspective, questionnaire data reflect long-term exposure factors (such as diet, smoking, and family history), while serological markers reflect the current physiological and pathological state of the gastric mucosa. The combination of these two allows for a more comprehensive characterization of the different stages of disease development, thereby improving predictive performance. This aligns with of “cost-effectiveness, social affordability, and public acceptance” the objectives screening (30, 31).

The results of this study show that both models exhibit high sensitivity (nearly 90% for both), suggesting strong capabilities for “minimizing missed diagnoses” in screening scenarios. This is particularly critical for the early identification of gastric cancer and precancerous lesions. Although the models’ specificity is relatively low, in screening strategies, high sensitivity takes precedence over high specificity, as it is more effective in identifying potential high-risk populations. Given their high sensitivity, these models may have potential utility as preliminary risk stratification tools in screening-oriented clinical settings, particularly where endoscopic resources are limited. In primary care or resource-constrained environments, questionnaire and serological assessment may help prioritize individuals at higher risk for referral to gastroscopy, thereby improving the efficiency of endoscopic resource utilization. Such an approach may also facilitate earlier identification of individuals at increased risk of gastric lesions before invasive examination. Nevertheless, further prospective validation in broader screening populations remains necessary.

Classification matrix analysis further suggested that the models preferentially identified individuals with clinically relevant abnormalities, while a subset of objectively normal individuals was categorized as high risk. Such false-positive classifications may increase the proportion of individuals referred for further endoscopic evaluation, potentially resulting in additional healthcare utilization and unnecessary diagnostic interventions in low-risk populations. Therefore, the present models may be more appropriately applied as risk stratification tools rather than standalone diagnostic methods, particularly when combined with serological indicators and clinical assessment to improve screening efficiency. Notably, the predictive performance of the models showed good concordance with serological findings and endoscopic pathological results, supporting their potential applicability and stability in real-world clinical practice. Consequently, these models may contribute to reducing non-selective screening approaches and improving the precision of high-risk population identification. Furthermore, although conventional discrimination metrics, including sensitivity, specificity, PPV, NPV, and AUC, were used to evaluate predictive performance in the present study, additional methodological assessments may further strengthen the evaluation of clinical utility. Future studies incorporating model calibration analyses, decision curve analysis (DCA), and net clinical benefit assessment may provide a more comprehensive understanding of model performance across different risk thresholds and support optimization for real-world implementation.

This study has certain limitations. First, the study population primarily consisted of patients presenting with gastrointestinal symptoms, which may introduce selection bias and limit the direct generalizability of the findings to asymptomatic population-based screening settings. In addition, this study was based on collected clinical data, which may be subject to inherent information bias. Although patients were enrolled from multiple hospitals, potential heterogeneity in patient characteristics and clinical practice patterns across participating centers may also have influenced model performance. Future prospective multicenter studies are warranted to further validate the robustness and generalizability of the models. Second, Although the present study primarily evaluated the performance of the established models using an expanded clinical cohort collected after model development, a limited proportion of early cases may have overlapped with the original development dataset due to the continuous accumulation of clinical data. Therefore, potential data overlap cannot be entirely excluded. Future studies using fully independent multicenter cohorts and temporal external validation are warranted to further confirm model robustness and generalizability. Furthermore, there remains room for optimization of the current predictive framework. Future studies may incorporate imaging, microbiome-related features, and artificial intelligence–assisted algorithms to further improve predictive performance. In addition, prospective cohort evaluation and external validation in independent populations are warranted to confirm model robustness and generalizability. Continuous refinement of predictive algorithms based on longitudinal follow-up data may further enhance clinical applicability.

In summary, the stepwise screening model developed in this study, integrating questionnaire and serological data, demonstrated favorable predictive performance and potential clinical utility in the risk assessment of gastric diseases. It provides a feasible approach for the early identification and stratified management of gastric organic lesions, with promising applicability in clinical screening settings.

## Data Availability

The original contributions presented in the study are included in the article/**Supplementary Material**. Further inquiries can be directed to the corresponding authors.

## Supplementary material

Supplementary Table 1 Gastric function prediction system questionnaire.

Supplementary Table 2 Definitions of normal and abnormal gastric function test results.

Supplementary Table 3 Definitions of normal and abnormal gastroscopy (including pathology) findings.
