# Supplementary material for: The clinical value of analyzing predictive models for gastric function and gastric organic lesions
Source: Front Endocrinol (Lausanne). 2026 Jun 15;17:1852014. doi: 10.3389/fendo.2026.1852014 (PMC13310679; doi:10.3389/fendo.2026.1852014)
Supplement: Supplementary Table 1 — Gastric function prediction system questionnaire. [file Table1.docx]

**Supplementary table 1**

Gastric Function Prediction System Questionnaire

**I. General Information**

Name: ID Number: Gender: □ Male; □ Female Age: years old;

Ethnicity: □ Han □ Yao □ Zhuang □ Hui □ Other (please specify)

Weight (unit: kg): Height (unit: cm): Occupation:

Educational Level: □ Illiterate; □ Elementary School; □ Junior High School; □ High School; □ College

Daily Language: □ Mandarin; □ Cantonese; □ Hakka; □ Chaoshan; □ Other

Permanent Residence: □ Urban Area; □ Town; □ Rural Area:

Phone: , ID Number:

Date of Visit: , ,

**II. Epidemiological Survey (Multiple selections allowed)**

1. Family history of esophageal cancer, gastric cancer, or colorectal cancer (e.g., first-degree relatives such as parents, brothers, or sisters with gastric cancer)

Esophageal cancer: □ Yes Age at diagnosis: □ No

Gastric cancer: □ Yes Age at diagnosis: □ No

Colorectal cancer: □ Yes Age at diagnosis: □ No

2. High-salt diet (daily salt intake greater than 10 grams, equivalent to one soda bottle cap) □ Yes □ No

3. Consumption of pickled foods □ Occasionally (less than three times per week) □ Frequently (at least three times per week)

4. Consumption of fried and smoked foods □ Occasionally (less than three times a week) □ Frequently (at least three times a week)

5. Fresh fruit intake □ Occasionally (less than three times a week) □ Frequently (at least three times a week)

6. Fresh vegetable intake □ Occasionally (less than three times a week) □ Frequently (at least three times a week)

7. Tea consumption □ Occasionally (less than three times a week) □ Frequently (at least three times a week)

7.1. Tea drinking temperature: □ Cold □ Warm □ Hot

7.2. Main types of tea consumed: □ Green tea □ Black tea □ Oolong tea □ Dark tea (Pu’er tea) □ Other

8. Smoking □ No

□ Yes: Previously smoked an average ofper day cigarettes for a total of years have been smoke-free for; years;

Current average daily smoking: cigarettes; total smoking duration : years.

9. Alcohol History □ None

□ Yes:

Type: □ High-proof alcohol (≥40°) □ Medium-proof alcohol (20°–40°) □ Low-proof alcohol (≤20°)

Alcohol intake: (ml/week)

Average weekly alcohol intake: g; Total alcohol consumption:years of

1. Water source □ Tap water (including bottled water, filtered water, etc.) □ Well water □ River/stream water

Do you drink hot water (water that is quite hot or scalding)? □ No □ Yes

11. Eating speed □ Fast □ Slow

12. Diabetes (FBS ≥ 7.0 mmol/L or 126 mg/dL) □ No □ Yes □ Not checked;

13. History of hypertension (BP ≥ 140/90 mmHg or taking antihypertensive medication) □ No □ Yes □ Not checked;

14. Hypertriglyceridemia (TG ≥ 1.7 mmol/L, or taking lipid-lowering medication) □ No □ Yes □ Not checked;

**III. Previous Examinations and Treatment History**

1. **H. pylori infection**: □ Not checked; □ Positive; □ Negative; Method: □ Tissue urease test, □ C13 breath test

D ate of initial Hp infection diagnosis: , Did you receive standard treatment: □ Yes □ No

Did the test result turn negative after treatment? (□ Yes, □ No, □ Not tested) Method: □ Tissue urease, □ C13 breath test

2. **Chronic atrophic gastritis**: □ Yes; □ No; □ Not tested, Initial diagnosis date:

Is there intestinal metaplasia? (□ Yes, □ No, □ Uncertain)

Is there dysplasia/intraepithelial neoplasia (□ Yes, □ No, □ Uncertain)

3. **Gastric Polyps**: □ Yes; □ No; □ Not checked; Initial Visit Date: Year Month

Biopsy performed (□ Yes, □ No), Pathological diagnosis [□ Adenoma, □ Inflammatory polyp, □ Dysplasia]

Was the polyp removed? (□ Yes, □ No), Date of removal: .

Post-resection pathological diagnosis [□ Adenoma, □ Inflammatory polyp, □ Dysplasia]

4. **Anemia**: □ Yes □ No □ Not checked

5. **Gastric ulcer**: □ Yes □ No □ Not checked Initial diagnosis date:

Ulcer stage: □ Active □ Healing □ Cicatrized

Comorbid atrophy: □ Yes, □ No; Comorbid intestinal metaplasia: □ Yes, □ No; Comorbid dysplasia: □ Yes, □ No

Date of last follow-up: , Result: □ Active phase, □ Healing phase, □ Scarring phase.

6. **Residual stomach after gastrectomy** □ Yes □ No Date of surgery:

Reason for surgery: □ Gastric ulcer (with bleeding), □ Gastric cancer, □ Other conditions.

Are regular follow-up gastroscopies performed (□ Yes, □ No)? Approximate interval between follow-up gastroscopies:

Date of most recent gastroscopy:

7. **Barrett’s esophagus:** □ Yes □ No □ Not examined Date of diagnosis: Year Month,

Accompanied by intestinal metaplasia (□ Yes, □ No, □ Uncertain), dysplasia (□ Yes, □ No, □ Uncertain)

8. **Endoscopic treatment** (□ Yes □ No Procedure: □ APC, □ EMR, □ ESD) Date of treatment:

Reason for endoscopic treatment: □ Gastric polyps □ Barrett’s esophagus □ Other

**IV. Major Symptoms in the Past 3 Months**

□ Abdominal pain; □ Abdominal distension; □ Nausea; □ Vomiting; ☑ Heartburn; □ Belching; □ Early satiety;

□ Postprandial discomfort; □ Heartburn; □ Melena; □ Weight loss; □ Loss of appetite; □ Dysphagia;

□ Discomfort behind the sternum; □ No significant symptoms;

**Supplementary table 2** Definitions of Normal and Abnormal Gastric Function Test Results

| Parameter | Normal | Abnormal |
| --- | --- | --- |
| Gastrin-17 | ≤5.7 pmol/L | >5.7 pmol/L, or <1 pmol/L |
| Hp Antibody | Negative | Positive |
| Pepsinogen I | 70 μg/L to 165 μg/L | >165 μg/L, or <70 μg/L |
| Gastrinogen II | < 11 μg/L | ≥11 μg/L |
| Pepsinogen I/II ratio | ≥7 | <7 |
| Hp-positive is also considered normalalone | | Abnormal gastric function:  (1) Hp(+). Must be accompanied by at least one additional abnormality to be defined as abnormal gastric function;  (2) Hp(–). Abnormal gastric function is defined by the presence of any one or more of the other abnormalities. |

**Supplementary table 3** Definitions of Normal and Abnormal Gastroscopy (including Pathology) Findings

| Criteria for Normal Gastroscopy (Including Pathology) (Absence of Organic Gastric Disease): | Chronic non-atrophic gastritis alone (mild/moderate, may be accompanied by bile reflux),  Only chronic superficial gastritis (mild to moderate, possibly accompanied by bile reflux). |
| --- | --- |
| Criteria for Abnormal Gastroscopy (Including Pathology) (Indicating Organic Gastric Disease) | The following organic lesions are classified as abnormalities:  Chronic non-atrophic gastritis (severe, with or without other endoscopic findings), chronic non-atrophic gastritis with erosions, chronic superficial gastritis with erosions, chronic atrophic gastritis, gastric ulcers, gastric polyps, with intestinal metaplasia, with dysplasia, intraepithelial neoplasia, early/advanced gastric cancer, and gastric cancer.  Chronic non-atrophic gastritis or chronic superficial gastritis, when accompanied by other diagnoses such as duodenal ulcer or esophagitis, is also considered abnormal. |
